# Confined placental mosaicism revisited: Impact on pregnancy characteristics and outcome

**DOI:** 10.1371/journal.pone.0195905

**Published:** 2018-04-12

**Authors:** Jérôme Toutain, Damien Goutte-Gattat, Jacques Horovitz, Robert Saura

**Affiliations:** 1 CHU de Bordeaux, Service de Génétique Médicale, Bordeaux, France; 2 Université de Bordeaux, Bordeaux, France; Mount Sinai Health System, CANADA

## Abstract

**Objectives:**

We wanted to re-evaluate the influence of confined placental mosaicism subtypes (type 2 and type 3) on pregnancy characteristics and outcome.

**Material and methods:**

From July 2009 to December 2015, 5512 chorionic villus samplings were performed in our Fetal Medicine Center. Conventional karyotyping was performed after long-term and short-term cultured villi to define type 2 or type 3 confined placental mosaicisms. Karyotype after amniocentesis was performed to exclude true fetal mosaicism, when appropriate. Pregnancy characteristics and outcomes were collected and compared to a control population.

**Results:**

Thirty-six (0.65%) confined placental mosaicisms were observed (13 type 2 and 23 type 3). Nuchal translucency was not increased for type 2 and type 3 confined placental mosaicisms. Pregnancy characteristics and outcomes were comparable between type 2 confined placental mosaicisms and the control population. In type 3 confined placental mosaicisms, median first trimester serum pregnancy-associated plasma protein A was lower than for the control population (p<0.001), preterm births were noticed in 56% (p<0.001), small for gestational age newborns in 74% (p<0.001), and adverse pregnancy outcome was reported in 35% (p<0.01).

**Conclusion:**

Although type 2 confined placental mosaicisms appeared to have no influence on pregnancy characteristics and outcome, type 3 confined placental mosaicisms were associated with low levels of first trimester serum pregnancy-associated plasma protein A, preterm birth, small for gestational age newborns, and adverse pregnancy outcomes.

## Introduction

Confined placental mosaicism (CPM) is defined as the presence of chromosomal abnormalities in the extra-embryonic tissue which are absent from the fetal tissue [1]. These chromosomal abnormalities are observed in about 1 to 2% of chorionic villus samplings (CVS) carried out for prenatal diagnosis between the 9^th^ and 12^th^ weeks of amenorrhea (weeks) [2]. Once identified, CPM can be classified into three subtypes (types 1, 2 and 3 CPM) according to the placental localization of the chromosomal abnormality [1]. In type 1 CPM (CPM1), the chromosomal abnormality is found exclusively in the cytotrophoblast (i.e. the chromosomal abnormality is observed only after examination of short-term culture villi (STC-villi)). For type 2 CPM (CPM2), the chromosomal abnormality is limited to the mesenchymal core of the chorionic villi (i.e. the chromosomal abnormality is observed only after examination of long-term culture villi (LTC-villi)). Type 3 CPM (CPM3) is defined as the presence of a chromosomal abnormality in both the cytotrophoblast and the mesenchymal core of the chorionic villi (i.e. the chromosomal abnormality is present after both STC-villi and LTC-villi analysis).

In 1983, Kalousek and Dill described CPM in human conceptions for the first time [1]. From their initial clinical cases, these authors had already suspected an association between CPM and intrauterine growth restriction. In 1992, Simoni and colleagues reported that a trisomy 16 confined to placental tissue had a negative effect on fetal growth and pregnancy outcome [3]. During the past 30 years, several studies have investigated the association between CPM involving trisomy 16 and other trisomies, and fetal growth, but conflicting results have been reported. For some authors, the outcome of pregnancies complicated by CPM appeared to be predominantly favorable [4–7]. For others, pregnancies complicated by CPM were associated with an increased risk of intrauterine growth restriction and/or intrauterine fetal death [8–12]. The objectives of this study were to re-evaluate the influence of CPM2 and CPM3 on pregnancy characteristics and outcome.

## Material and methods

### Study design

This retrospective monocentric study was based on 5512 patients who underwent invasive prenatal diagnosis by CVS between July 2009 and December 2015 in the Fetal Medicine Center of Bordeaux University Hospital (France). This study was approved by the Institutional Review Board of Bordeaux University Hospital (France).

### Sampling procedure and cytogenetic examination of chorionic villi

The sampling procedure and cytogenetic examination of chorionic villi was performed as previously described [12]. Briefly, extra-amniotic transabdominal sampling of the chorionic villi was carried out from the 12^th^ week by an experienced practitioner [13]. After an extemporaneous examination of the chorionic villi by the cytogeneticist, they were divided into three unequal parts:

1. About 5 mg of chorionic villi were used to search for main aneuploidies using a rapid (24-hour) fluorescence *in situ* hybridization technique applied to mesenchymal core cells, after specific enzymatic dissociation [14],
2. The majority of the sample (usually up to 20 mg) was used for LTC-villi and conventional karyotyping was established after Giemsa staining and heat denaturation (‘R-bands’); at least 16 mitoses were analyzed. Mosaic placental tetraploidies were excluded from the study,
3. The rest of the sample (~ 5 mg) were prepared and conserved until a later possible cytogenetic examination of the cytotrophoblast by STC-villi examination. STC-villi was not systematically perform in our Center, but it could be achieved retrospectively:
  - when a large amount of decidua was observed when villi were sorted under a binocular microscope, in a fetus with a chromosome formula 46,XX after LTC;
  - when mosaicism was observed after LTC,
  - or in the case of CPM, to characterize CPM subtypes (CPM2 or CPM3) [12].

### Patient information and karyotype after amniocentesis

Patients were always informed of the abnormal cytogenetic result after CVS. Based on the risk of diagnosing a true fetal mosaicism and in accordance with the patients’ choice, a karyotype after amniocentesis could therefore have been performed in the following cases:

- Testing for uniparental disomy for CPM involving trisomies 14 or 15 [15], with confirmation of the fetal chromosomal formula by karyotyping,
- Excluding true fetal mosaicism, when the placental chromosomal abnormality involved a potentially viable chromosomal abnormality, as in the case of placental mosaicism for trisomies 8, 9, 13, 18, 21 and monosomy X, in the absence of fetal structural ultrasound abnormalities [12, 16],
- Patient anxiety, despite the reassuring information provided on the very low risk of true fetal mosaicism regarding some trisomies, in the absence of fetal structural ultrasound abnormalities validated by an experienced practitioner (for example in the case of placental trisomy 2, 4, 5, 7, 10, 12, 16, or 22) [12, 16].

Conventional karyotyping after amniocentesis was then performed from at least 16 mitoses from multiple colonies derived from at least three coverslips.

### Control population

A control population was randomly chosen from patients who were referred during the same study period (July 2009 and December 2015) to our Fetal Medicine Center for an invasive prenatal procedure by CVS, and in whom conventional karyotyping was strictly normal after LTC-villi examination [17]. This control population was at least twice as large as the number of patients with CPM [18].

### Pregnancy outcome

Preterm and very preterm births were defined as birth before 37 and 33 weeks, respectively [19]. Small for gestational age (SGA) and severe SGA newborns were defined when birth weight was below the 10^th^ percentile and the 3^rd^ percentile, respectively [20, 21]. In this study, adverse pregnancy outcome was defined as the occurrence of intrauterine fetal death, very preterm birth, severe SGA newborns, or perinatal death.

### Statistics

Qualitative data (expressed as percentages) were compared using the Fischer exact test or the Chi-square test, whichever was appropriate. Quantitative data expressed as ‘median, quartile 1 –quartile 3’ were compared using the Mann-Whitney test; and when expressed as ‘mean ± standard deviation’, data were compared using the Student-t test. Association between quantitative data was studied using the Pearson correlation test.

## Results

### Confined placental mosaicism

**Fig 1:**
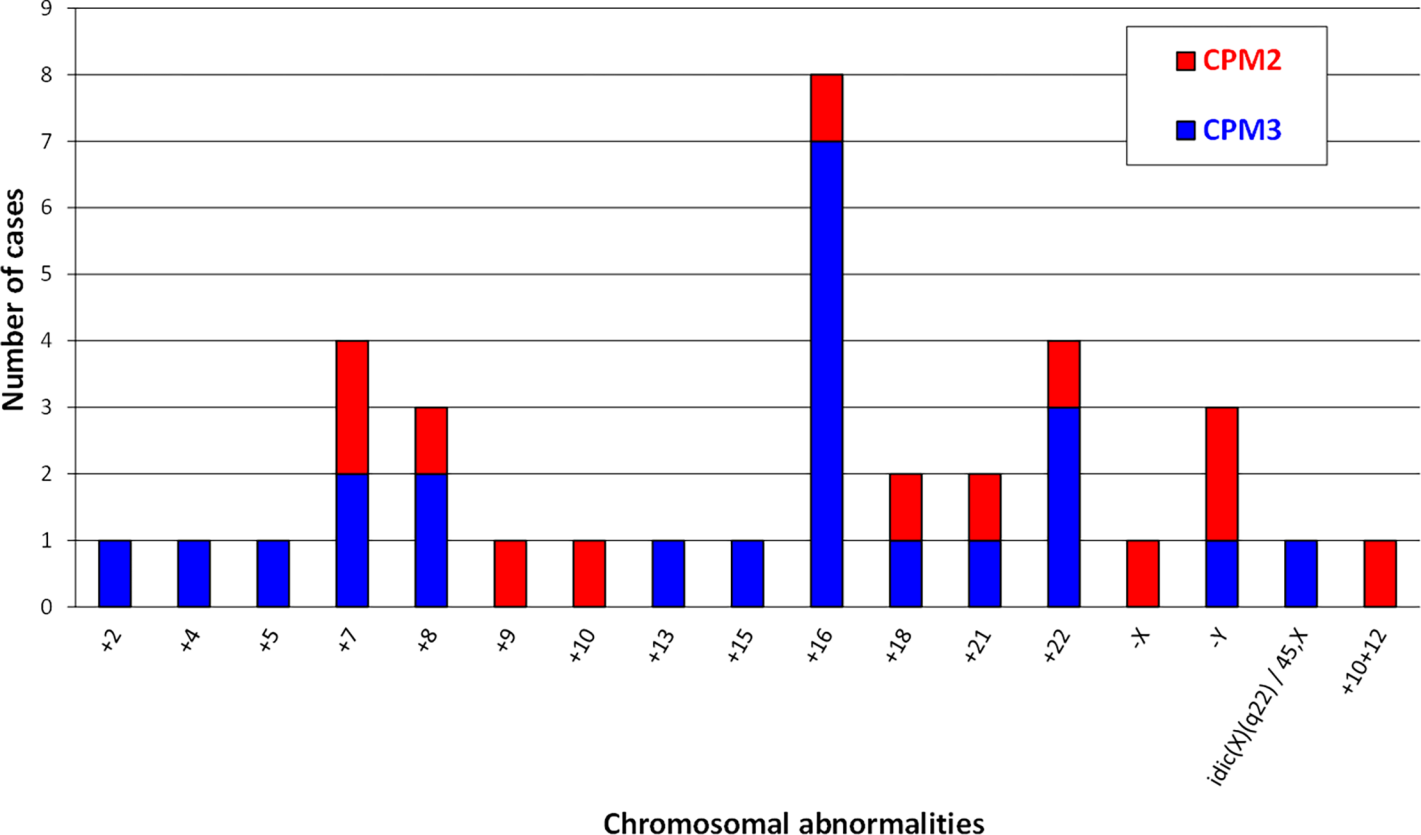
Distribution of chromosomal abnormalities observed in 36 confined placental mosaicisms (CPM) (CPM2: Type 2 CPM; CPM3: Type 3 CPM).

Among 5512 CVS, there were 36 (0.65%) CPM2-3 cases, including 13 CPM2 and 23 CPM3 (Fig 1, details in S1 Table). LTC-villi revealed a full (non-mosaic) trisomy in mesenchymal core cells in 23% (3/13) of CPM2 and in 61% (14/23) of CPM3 (S1 Table).

### Prenatal diagnosis indications

No differences were observed in the reason for CVS between the control, CPM2 and CPM3 groups, with the exception of CPM3 being more frequent in CVS performed because an abnormal ‘First trimester combined test for Down syndrome’ (65% of patients with CPM3 were referred for this indication, p<0.05) (Table 1).

**Table 1:** Influence of type 2 and type 3 confined placental mosaicisms on pregnancy characteristics and outcome.

Pregnancy characteristics	Control population (n = 93)	CPM2 and CPM3 (n = 36)	P value	CPM2 (n = 13)	P value	CPM3 (n = 23)	P value
Maternal age (years) (mean ± sd)	34 ± 6	34 ± 6	NS	35 ± 6	NS	34 ± 6	NS
Prenatal diagnosis indication (n, %):							
*- Maternal age*	12 (13)	4 (11)	NS	2 (15)	NS	2 (9)	NS
*- Antecedent*	15 (16)	3 (8)	NS	2 (15)	NS	1 (4)	NS
*-First trimester combined test*	33 (35)	19 (53)	NS	4 (31)	NS	15 (65)	<0.05
*- Second trimester maternal serum screening*	8 (9)	4 (11)	NS	3 (23)	NS	1 (4)	NS
Nuchal translucency (mm) (mean ± sd)	1.7 ± 0.9	1.3 ± 0.6	<0.01	1.5 ± 0.7	NS	1.2 ± 0.5	<0.01
First-trimester free β-HCG (MoM) [median (Q1-Q3)]	1.66 (0.88–2.78)	1.46 (0.88–2.45)	NS	1.18 (0.69–2.23)	NS	1.69 (0.99–2.41)	NS
First-trimester PAPP-A (MoM) [median (Q1-Q3)]	0.76 (0.48–1.20)	0.26 (0.16–0.63)	<0.001	0.63 (0.32–0.84)	NS	0.19 (0.11–0.39)	<0.001
Chorionic villus sampling tern (weeks) [median (Q1-Q3)]	14 (13–19)	14 (14–20)	NS	16 (14–22)	NS	14 (13–17)	NS
Gestation age (weeks) (mean ± sd)	39 ± 2	36 ± 4	<0.001	38 ± 3	0.06	35 ± 4	<0.001
Preterm birth (n, %)	7/83 (8)	13/29 (45)	<0.001	3/11 (27)	NS	10/18 (56)	<0.001
Very preterm birth (n, %)	1/83 (1)	6/29 (21)	<0.01	1/11 (9)	NS	5/18 (28)	<0.001
SGA newborns (n, %)	14/82 (17)	16/30 (53)	<0.001	2/11 (18)	NS	14/19 (74)	<0.001
Severe SGA newborns (n, %)	5/82 (6)	7/30 (23)	<0.05	0 (0)	-	7/19 (37)	<0.01
Adverse pregnancy outcome (n, %)	8/84 (10)	4/36 (11)	NS	1/13 (8)	NS	8/23 (35)	<0.01

β-HCG: beta-human chorionic gonadotropin; CPM2: type 2 confined placental mosaicism; CPM3: type 3 confined placental mosaicism; MoM: multiple of the median; NS: non significant; PAPP-A: pregnancy-associated plasma protein A; Q1: quartile 1; Q3: quartile 3; sd: standard deviation; SGA: small for gestational age; weeks: weeks of amenorrhea.

### Nuchal translucency

Mean nuchal translucency (NT) measurement in the first trimester was significantly higher in the control population than in CPM2-3 cases, for whom NT was in a normal range (Table 1). In the control population, 17% (15/88) fetuses had an NT above the 95^th^ percentile, whereas this was the case for 6% (2/33) in CPM2-3 cases (2 fetuses with CPM2, no fetus with CPM3).

### First trimester combined test for Down syndrome

**Fig 2:**
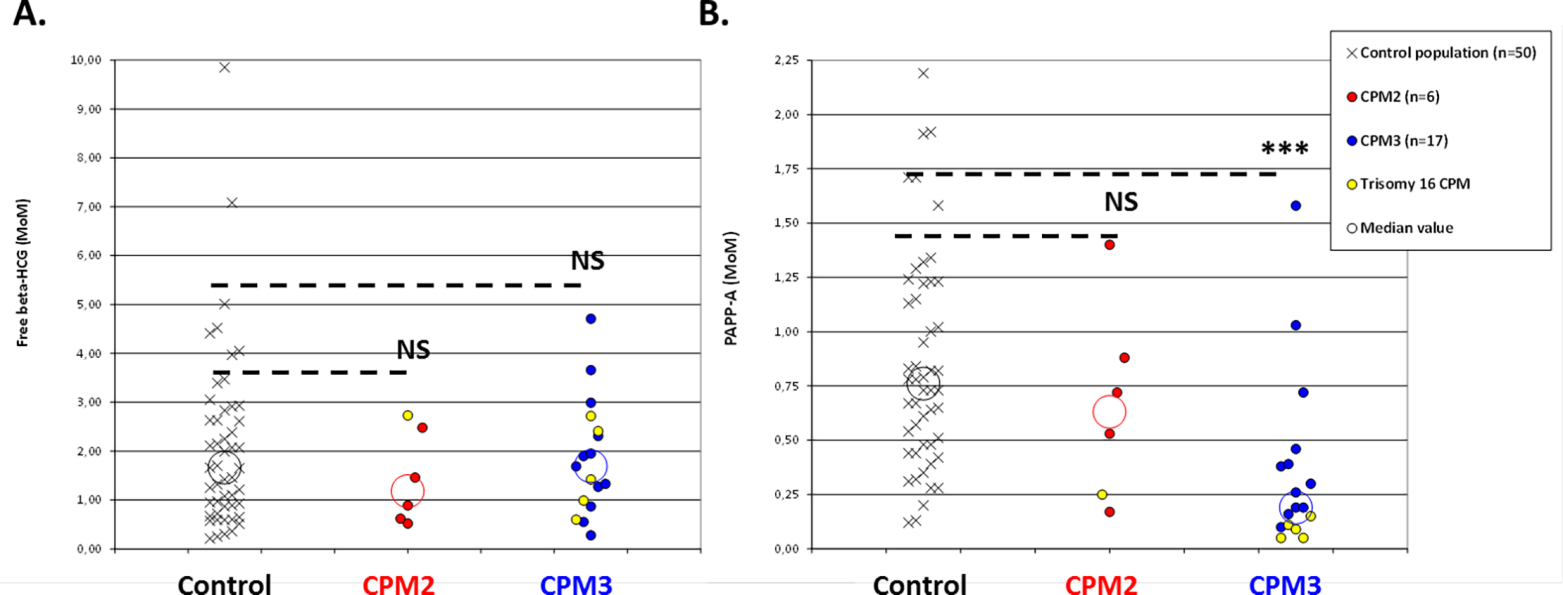
First trimester free beta-human chorionic gonadotropin and serum pregnancy-associated plasma protein A levels, and confined placental mosaicisms (CPM). A. Distribution of first trimester free beta-human chorionic gonadotropin (β-HCG) for the control population, type 2 confined placental mosaicisms (CPM2) and type 3 confined placental mosaicisms (CPM3). B. Distribution of first trimester serum pregnancy-associated plasma protein A (PAPP-A) for the control population, CPM2 and CPM3 (***: p<0.001; MoM: multiple of the median; NS: non significant).

No differences were observed in the levels of free beta-human chorionic gonadotropin (free β-HCG) between the control, CPM2 and CPM3 groups (Table 1, Fig 2A).

For first trimester serum pregnancy-associated plasma protein A (PAPP-A) levels, the median value was lower for CPM3, compared to CPM2 and the control population (Table 1, Fig 2B). More precisely, 71% (12/17) of patients with CPM3 had a first trimester PAPP-A level below the 5^th^ percentile (i.e. <0.45 multiple of the median (MoM)), and 53% (9/17) had a PAPP-A level below the 1^st^ percentile (i.e. < 0.29 MoM) [22]. For the six cases of trisomy 16 restricted to the placenta, PAPP-A levels were always below the 1^st^ percentile (Fig 2B, ‘yellow symbols’).

### Pregnancy outcome

**Fig 3:**
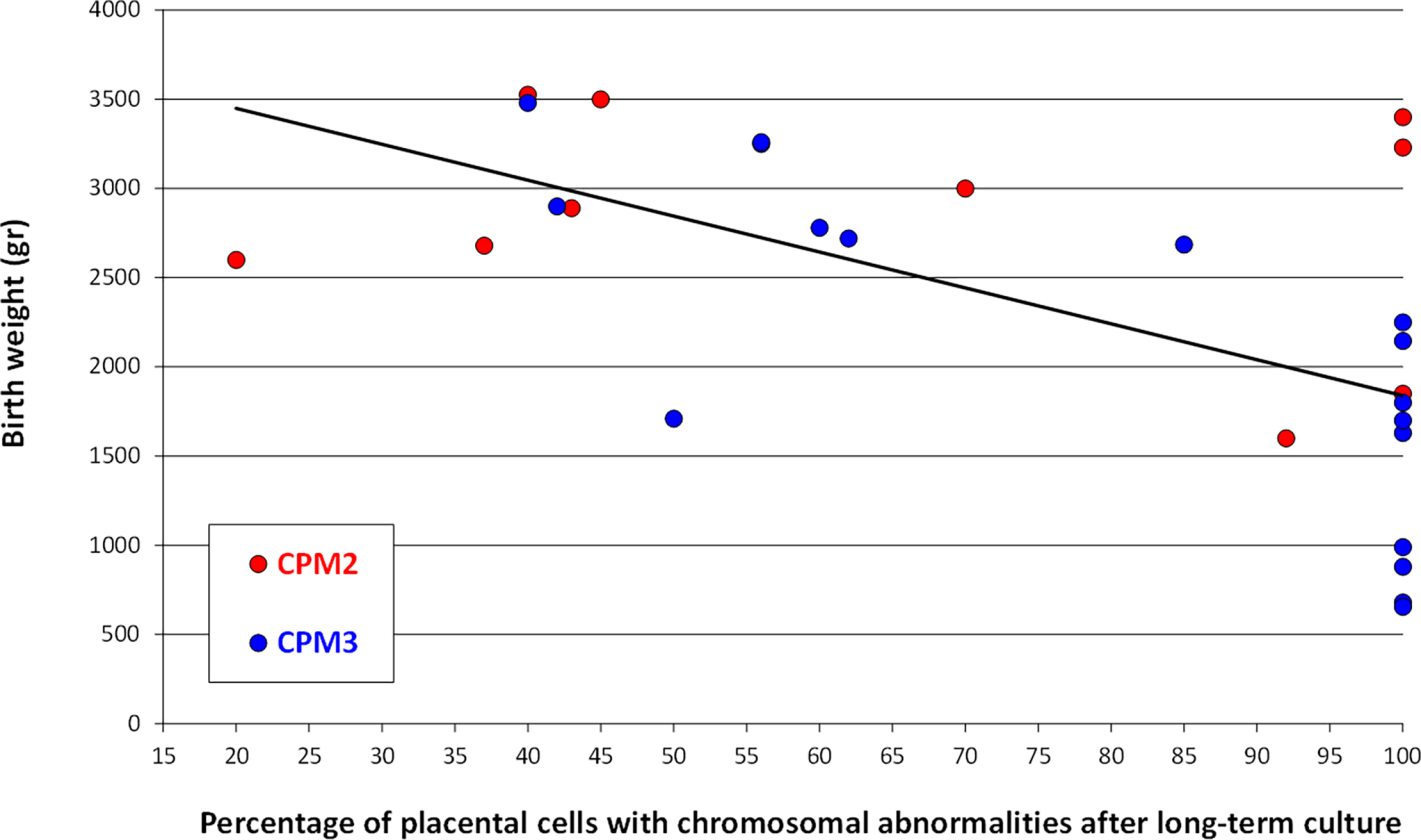
Association between birth weight and percentage of placental cells with chromosomal abnormalities after long-term cultured villi for type 2 confined placental mosaicisms (CPM2) and type 3 confined placental mosaicisms (CPM3) (Pearson's correlation coefficient = -0.61, p<0.001).

In CPM2-3 cases, the term of the pregnancy occurred earlier, and SGA newborns were higher, compared to the control population (Table 1). Considering CPM2 separately, the occurrence of preterm birth and SGA newborns were similar to the control population (Table 1). Regarding CPM3, they were statistically associated with preterm birth (56%, p<0.001), very preterm birth (28%, p<0.001), SGA newborns (74%, p<0.001), severe SGA newborns (37%, p<0.01), and adverse pregnancy outcome (35%, p<0.01) (Table 1).The percentage of placental cells with chromosomal abnormalities after LTC-villi was negatively associated with birth weight (Pearson's correlation coefficient = -0.61, p<0.001) (Fig 3).

If we pool together data from our first study in the field (‘study #1’, published in 2010 [12]) with the data from the present study (‘study #2’), we are able to present 93 CPM2 and CPM3, distributed as follows:

- 50 CPM2 (study #1: 37, study #2: 13 CPM2),
- 43 CPM3 (study #1: 20, study #2: 23 CPM3) [12].

**Fig 4:**
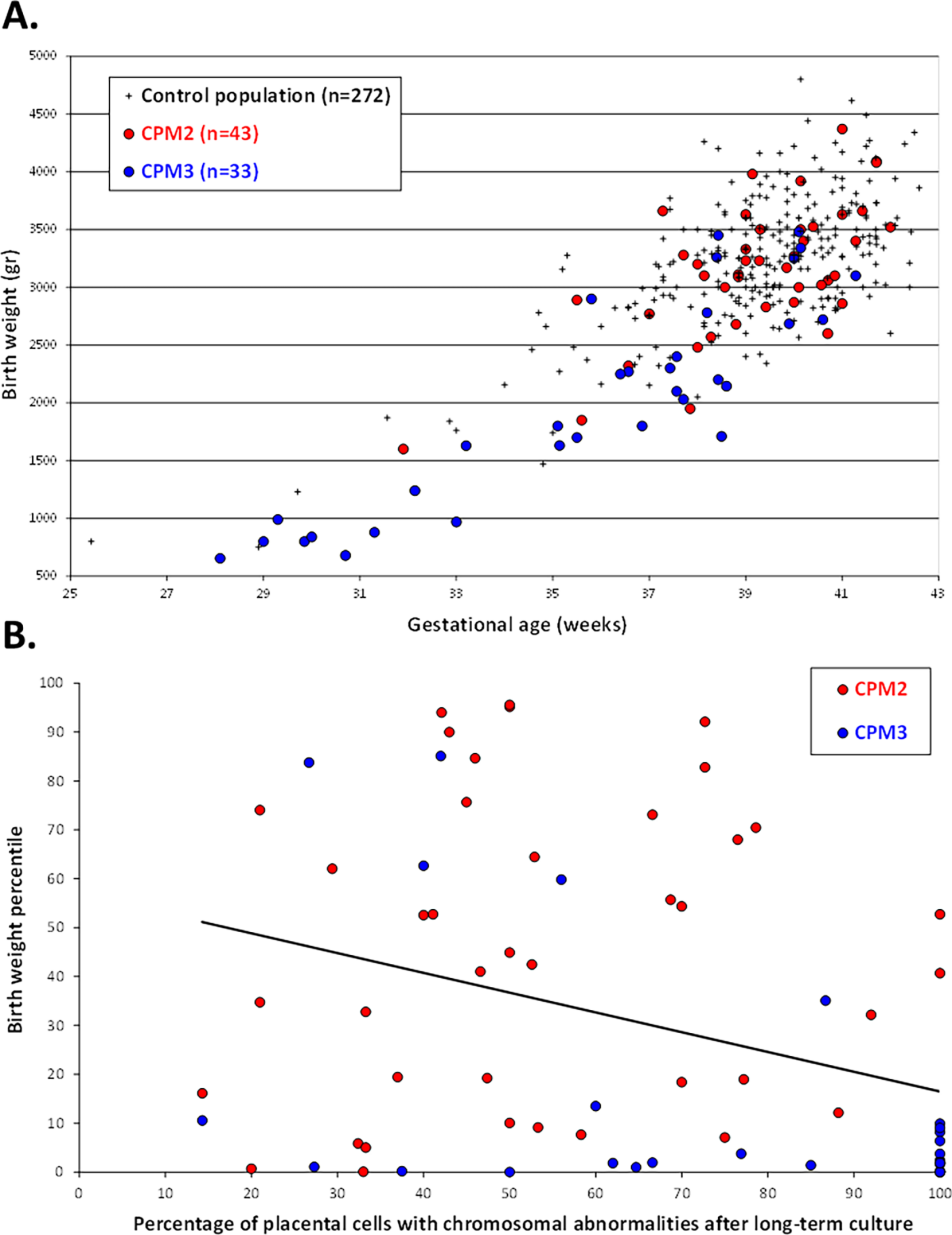
Type 2 confined placental mosaicisms (CPM2) and type 3 confined placental mosaicisms (CPM3) diagnosed in the fetal medicine center of the University Hospital of Bordeaux (France) from 19321 chorionic villus samplings performed between 1997 and 2015. A. Weight and gestation age at delivery for CPM2 and CPM3, and a control population. B. Association between birth weight percentile and percentage of placental cells with chromosomal abnormalities after long-term cultured villi for CPM2 and CPM3 (Pearson's correlation coefficient = -0.34, p<0.01).

These CPM were observed in 19321 patients (study #1: 13809, study #2: 5512 patients), who were referred to our Fetal Medicine Center between 1997 and 2015. As an illustration, Fig 4A depicts birth weight according to gestational age in these patients. In CPM2, 16% (7/43) of newborns were SGA (non-significant versus the control population, with 14% (38/272) SGA newborns), whereas in CPM3, 76% (25/33) were identified as SGA newborns (p<0.001), and 42% (14/33) as severe SGA newborns (p<0.001). Fig 4B illustrates the negative association between birth weight percentile and percentage of placental cells with chromosomal abnormalities after LTC-villi in these patients (Pearson's correlation coefficient = -0.34, p<0.01).

## Discussion

In 2010, our group reported a large retrospective monocentric study, based on 13809 CVS, specifically to test the influence of CPM subtypes on pregnancy outcome [12]. Our report demonstrated that CPM3 were clearly associated with preterm births, low birth weights and adverse pregnancy outcomes, while CPM2 had no effect on fetal development. However, the influence of CPM subtypes on fetal growth remained a controversial topic [23, 24]. In the present study, we confirm that CPM2 had no influence on fetal development. In contrast, pregnancies with CPM3 were associated with preterm births, SGA newborns and adverse pregnancy outcomes. We are therefore in agreement with authors for whom CPM of meiotic origin (mainly CPM3) is associated with an increased risk of intrauterine growth restriction and SGA newborns [9, 25].

Interestingly, we observed a negative association between birth weight and percentage of placental cells with chromosomal abnormalities after LTC-villi. Previously, Sifakis and colleagues reported a similar association in the case of trisomy 2 confined to placenta [26]. Our results therefore suggest that this negative association should be extended to the other trisomies restricted to the placenta. Our results are also in agreement with those recently reported for rare autosomal trisomies (“RATs”) revealed by whole-genome sequencing of maternal plasma cell-free DNA, suggesting a more likely outcome of CPM when the calculated trisomic fraction is much lower than the fetal fraction (theoretically in the case of low level placental mosaicism) [27].

One limitation of our study was the exclusion of CPM1, since we have not systematically established STC-villi for more than fifteen years now [12, 14]. STC-villi examination is based on cytotrophoblast cells, which do not seem to us, and to others, to be a reliable tissue to establish rapid fetal chromosomal formula [2, 14]. Since 1997, we have therefore abandoned the systematic use of STC-villi, and we now prefer to examine mesenchymal cells by a rapid fluorescence *in situ* hybridization technique, after specific enzymatic dissociation of the chorionic villi, for a reliable diagnosis of the main aneuploidies [14]. Since the introduction of non-invasive prenatal testing, using the cell-free DNA in maternal plasma, it is now well established that cytogenetic results obtained from cytotrophoblast cells must be considered as a screening test, not as a diagnosis [28]. Regarding CPM1 influence on fetal growth, in our experience of more than ten years using STC-villi (from 1984 to 1997), we did not notice any association between CPM1 and growth restriction (unpublished data). Interestingly, Baffero and colleagues identified 15% of SGA newborns in 52 patients with CPM1, which corresponds to the expected rate of SGA newborns in a prenatal diagnosis population (17% of SGA newborns in the control population of our study) [7].

In the first trimester, very low levels of PAPP-A are a well-defined condition for trisomy 16, as well as for trisomies 13, 18 and 21 [29]. Regarding other trisomies (such as those confined to the placenta), only few data are available for PAPP-A levels in the first trimester [30]. As expected, in our study, first trimester PAPP-A values for CPM involving trisomy 16 collapsed, falling below the 1^st^ percentile in each of the 6 cases observed [29]. Interestingly, we also observed that first trimester PAPP-A levels were lower in cases of CPM3 involving other trisomies than trisomy 16. In 58% of patients with CPM3 other than trisomy 16, first trimester PAPP-A levels were below the 5^th^ percentile. These levels can obviously not be considered as collapsed, but they were clearly below normal. This can presumably account for the large proportion of patients with CPM3 who were referred for prenatal diagnosis for ‘First trimester combined test for Down syndrome’. For CPM2, we did not identify any difference in PAPP-A levels with the control population, although it has to be mentioned that first trimester maternal serum markers were only available for 6 patients with CPM2. Further investigations on a larger cohort of patients would be needed to clarify the association between CPM subtypes and first trimester PAPP-A levels.

In conclusion, our study confirmed that a precise characterization of CPM subtypes needs to be carefully established. Although CPM2 had no effect on fetal development, CPM3 was associated with preterm birth, SGA newborns, and adverse pregnancy outcomes. The association of a low level of first trimester PAPP-A (below the 5^th^ percentile) with an isolated intrauterine growth restriction could also lead us to consider carrying out choriocentesis, rather than amniocentesis, in order to screen for CPM3.

## Supporting information

S1 Table Main characteristics of type 2 and type 3 confined placental mosaicisms observed in the study. (DOC)
