# Supplementary material for: Confined placental mosaicism revisited: Impact on pregnancy characteristics and outcome
Source: PLoS One. 2018 Apr 12;13(4):e0195905. doi: 10.1371/journal.pone.0195905 (PMC5897023; doi:10.1371/journal.pone.0195905)
Supplement: S1 Table — (DOC) [file pone.0195905.s001.doc]

| *Patients* | *Maternal*  *age* | *Nuchal*  *Translucency*  *(mm)* | *First-trimester*  *PAPP-A*  *(MoM)* | *First-trimester*  *free β-HCG (MoM)* | *Prenatal diagnosis*  *indication* | *CVS term*  *(weeks)* | *Tissue* | *CVS*  *karyotyping* | *CPM*  *subtype* | *Amniocentesis karyotyping* | *Birth*  *(weeks)* | *Birth weight*  *(gr)* | *Birth weight*  *(centile)* | *Comments* |
| --- | --- | --- | --- | --- | --- | --- | --- | --- | --- | --- | --- | --- | --- | --- |
| 1 | 39 | 1.2 | N/A | N/A | 1^st^ TCT | 13.6 | Mes  Cyt | 47,XY,+18  46,XY,+18 | 3 | 46,XY | _ | _ | _ | Absence of AFUF  - IUFD at 28 weeks |
| 2 | 39 | 0.7 | _ | _ | Maternal age | 13.4 | Mes  Cyt | 46,Xidic(X)(q22)[8]/46,XX[12]  45,X | 3 | 46,XX | 40.0 | 3480 | > 10^th^ | _ |
| 3 | 37 | N/A | _ | _ | 2^nd^ MSS | 18.7 | Mes  Cyt | 47,XY,+21[16]/46,XY[8]  46,XY | 2 | 46,XY | _ | _ | _ | TOP at 23.6 weeks requested by the couple (performed abroad), despite a reassuring genetic counseling based on a normal chromosomal formula at amniocentesis and the absence of AFUF |
| 4 | 35 | 1.4 | N/A | N/A | 1^st^ TCT | 13.4 | Mes  Cyt | 47,XX,+2[13]/46,XX[12]  47,XX,+2 | 3 | - | 38.3 | 1710 | < 3^rd^ | _ |
| 5 | 40 | 0.8 | 0.19 | 1.95 | Maternal age | 14.7 | Mes  Cyt | 47,XX,+7[17]/46,XX[8]  46,XX | 2 | - | 40.0 | 3000 | > 10^th^ | _ |
| 6 | 37 | 1.2 | _ | _ | 2^nd^ MSS | 22.6 | Mes  Cyt | 47,XX,+8[9]/46,XX[16]  46,XX | 2 | 46,XX | 38.0 | 2680 | > 10^th^ | _ |
| 7 | 37 | 1 | 0.88 | 0.89 | 1^st^ TCT | 14.9 | Mes  Cyt | 45,X[15]/46,XY[10]  45,X | 3 | 46,XY | 40.1 | 2720 | < 10^th^ | _ |
| 8 | 27 | N/A | _ | _ | ATCD  (Huntington’s disease) | 12.3 | Mes  Cyt | 47,XX,+9[14]/46,XX[11]  46,XX | 2 | - | _ | _ | _ | TOP - Fetus with Huntington’s disease |
| 9 | 29 | 0.9 | _ | _ | 2^nd^ MSS | 18.0 | Mes  Cyt | 47,XX,+16  47,XX,+16 | 3 | - | 38.6 | 2145 | < 3^rd^ | _ |
| 10 | 40 | 1.4 | N/A | N/A | AFUF (IUGR) | 24.0 | Mes  Cyt | 47,XY,+15  47,XY,+15 | 3 | 46,XY | 28.7 | 740 | < 3^rd^ | Biparental inherence of chromosomes 15 – Perinatal death at 25 days |
| 11 | 29 | 1.7 | 0.19 | 1.90 | 1^st^ TCT | 13.9 | Mes  Cyt | 45,X[10]/46,XY[15]  45,X | 2 | 46,XY | 40.1 | 3525 | > 10^th^ | _ |
| 12 | 43 | 0.9 | 0.72 | 2.99 | 1^st^ TCT | 13.4 | Mes  Cyt | 47,XY,+16  47,XY,+16 | 3 | - | 30.7 | 680 | < 3^rd^ | Good clinical outcome at 4 months |
| 13 | 39 | 1.0 | 0.26 | 1.69 | 1^st^ TCT | 15.4 | Mes  Cyt | 47,XY,+16[11]/46,XY[14]  47,XY,+16 | 3 | - | 35.0 | 2900 | > 10^th^ | _ |
| 14 | 44 | 0.6 | 0.39 | 0.87 | ATCD  (trisomy 21) | 13.0 | Mes  Cyt | 47,XX,+7[12]/46,XX[13]  46,XX | 2 | - | 39.4 | 3500 | > 10^th^ | _ |
| 15 | 44 | 0.8 | 0.05 | 2.41 | 1^st^ TCT | 13.9 | Mes  Cyt | 47,XY,+8[21]/46,XY[4]  47,XY,+8[9]/46,XY[5] | 3 | 46,XY | 39.9 | 2685 | < 10^th^ | _ |
| 16 | 32 | 0.9 | 0.05 | 0.60 | AFUF (IUGR) | 30.4 | Mes  Cyt | 45,X[23]/46,XY[2]  45,XY | 2 | 46,XY | 31.7 | 1600 | > 10^th^ | _ |
| 17 | 23 | 0.9 | 0.15 | 2.72 | 1^st^ TCT | 23.0 | Mes  Cyt | 47,XX,+18[10]/46,XX[13]  46,XX | 2 | 46,XX | 35.0 | 2890 | > 10^th^ | _ |
| 18 | 25 | 1.0 | 0.09 | 1.42 | ATCD  (Turner syndrome) | 13.1 | Mes  Cyt | 47,XY,+16  47,XY,+16 | 3 | - | 36.6 | 2250 | < 10^th^ | _ |
| 19 | 39 | 1.4 | 0.11 | 0.99 | Maternal age | 14.4 | Mes  Cyt | 47,XY,+8[4]/46,XY[16]  47,XY,+8[9]/46,XY[5] | 3 | 46,XY | N/A | N/A | N/A | _ |
| 20 | 25 | 1.7 | 0.25 | 2.73 | 1^st^ TCT | 14.4 | Mes  Cyt | 47,XY,+7[11]/46,XY[9]  47,XY,+7[2]/46,XY[10] | 3 | - | 40.1 | 3250 | > 10^th^ | _ |
| 21 | 40 | 1.1 | 0.10 | 0.28 | 1^st^ TCT | 14.3 | Mes  Cyt | 47,XX,+16  47,XX,+16 | 3 | - | _ | _ | _ | IUFD at 22.7 weeks - Fetal autopsy revealed  isolated IUGR |
| 22 | 27 | 3.0 | 1.40 | 2.48 | AFUF  (IUGR) | 25.0 | Mes  Cyt | 47,XY,+13  47,XY,+13 | 3 | 46,XY | _ | _ | _ | IUFD at 24.0 weeks |
| 23 | 40 | 2.5 | 0.30 | 1.27 | 1^st^ TCT | 14.7 | Mes  Cyt | 47,XX,+22  46,XX | 2 | - | 35.6 | 1850 | < 10^th^ | _ |
| 24 | 41 | 1.1 | 0.38 | 4.71 | Maternal age | 15.9 | Mes  Cyt | 48,XY,+10,+12  46,XY | 2 | - | 40.3 | 3350 | > 10^th^ | _ |
| 25 | 30 | 1.3 | 0.46 | 2.31 | AFUF  (cardiopathy) | 23.4 | Mes  Cyt | 47,XX,+5  47,XX,+5 | 3 | - | 33.7 | 1630 | < 10^th^ | Surgery of a ventricular septal defect  - Good clinical outcome – Postnatal cytogenetic examinations were normal (conventional karyotyping and array-CGH on blood lymphocytes and on cutaneous fibroblasts) |
| 26 | 31 | 1.5 | 0.53 | 1.46 | 1^st^ TCT | 15.0 | Mes  Cyt | 47,XX,+16  47,XX,+16 | 3 | - | 28.1 | 655 | < 3^rd^ | Good clinical outcome at 3 months |
| 27 | 26 | 2.2 | 1.58 | 3.66 | 1^st^ TCT | 15.1 | Mes  Cyt | 47,XX,+21[15]/46,XX[10]  47,XX,+21[8]/46,XX[8] | 3 | 46,XY | 38.6 | 2780 | > 10^th^ | _ |
| 28 | 35 | 1.5 | 0.16 | 1.33 | 1^st^ TCT | 13.6 | Mes  Cyt | 47,XY,+16  47,XY,+16 | 3 | - | 31.6 | 880 | < 3^rd^ | _ |
| 29 | 34 | 2.6 | 0.17 | 0.52 | 1^st^ TCT | 16.7 | Mes  Cyt | 47,XY,+16[4]/46,XY[16]  46,XY | 2 | - | 40.0 | 2600 | < 10^th^ | _ |
| 30 | 35 | 1.3 | N/A | N/A | 1^st^ TCT | 14.7 | Mes  Cyt | 47,XY,+22[14]/46,XY[11]  47,XY,+22 | 3 | - | 38.1 | 3260 | > 10^th^ | Pre-eclampsia |
| 31 | 37 | 1.2 | _ | _ | 2^nd^ MSS | 17.9 | Mes  Cyt | 45,X[6]/46,XY[14]  45,XY | 2 | 46,XY | 37.4 | 2600 | > 10^th^ | _ |
| 32 | 38 | 1.1 | N/A | N/A | 1^st^ TCT | 14.3 | Mes  Cyt | 47,XY,+22  47,XY,+22[2]/46,XY[18] | 3 | - | 38.1 | 2400 | < 10^th^ | _ |
| 33 | 32 | N/A | N/A | N/A | 1^st^ TCT | 22.1 | Mes  Cyt | 47,XY,+7  47,XY,+7 | 3 | 46,XY | 35.7 | 1700 | < 3^rd^ | Biparental inherence of chromosomes 7 - Pre-eclampsia |
| 34 | 25 | 0.7 | N/A | N/A | AFUF  (short fetal nasal bone length) | 25.9 | Mes  Cyt | 47,XX,+4  47,XX,+4 | 3 | - | 29.9 | 990 | < 10^th^ | _ |
| 35 | 29 | 1.1 | 1.03 | 0.55 | AFUF  (short fetal nasal bone length) | 24.4 | Mes  Cyt | 47,XX,+10  46,XX | 2 | - | 39.0 | 3230 | > 10^th^ | _ |
| 36 | 38 | 0.8 | 0.72 | 0.62 | 1^st^ TCT | 14.3 | Mes  Cyt | 47,XX,+22  47,XX,+22 | 3 | - | 35.3 | 1800 | < 10^th^ | _ |
| 1^st^ TCT = 1^st^ trimester combined test; 2^nd^ MSS = 2^nd^ trimester maternal serum screening; AFUF = abnormal fetal ultrasound findings; ATCD = antecedent; CGH = comparative genomic hybridization; CPM = confined placental mosaicism; CVS = chorionic villus sampling; Cyt = cytotrophoblast; IUFD = intra-uterine fetal death; IUGR = intrauterine growth restriction; Mes = mesenchymal core; MoM: multiple of the median; N/A = data not available; TOP = termination of pregnancy; UPD = uniparental disomy; weeks = weeks of amenorrhea. | | | | | | | | | | | | |  |  |
